# Toxicology Effects of Cadmium in *Pomacea canaliculate*: Accumulation, Oxidative Stress, Microbial Community, and Transcriptome Analysis

**DOI:** 10.3390/ijms26020751

**Published:** 2025-01-17

**Authors:** Mingxin Qiu, Xiaoyang Bi, Yuanyang Liu, Huashou Li, Dongqin Li, Guikui Chen

**Affiliations:** 1 Guangdong Laboratory for Lingnan Modern Agriculture, Guangdong Provincial Key Laboratory of Agricultural & Rural Pollution Abatement and Environmental Safety, College of Natural Resources and Environment, South China Agricultural University, Guangzhou 510642, China; 20222005009@stu.scau.edu.cn (M.Q.); 20221005001@stu.scau.edu.cn (X.B.); 1260710657@stu.scau.edu.cn (Y.L.); lihuashou@scau.edu.cn (H.L.); 2 Institute of Quality Standard and Monitoring Technology for Agro-Products, Guangdong Academy of Agricultural Sciences, Guangzhou 501640, China

**Keywords:** *Pomacea canaliculate*, cadmium, oxidative stress, gut microbiota, transcriptome

## Abstract

Cadmium (Cd) pollution poses an important problem, but limited information is available about the toxicology effects of Cd on freshwater invertebrates. We investigated the accumulation, oxidative stress, microbial community changes, and transcriptomic alterations in apple snails *(Pomacea canaliculata*) under Cd stress. The snails were exposed to the 10 μg/L Cd solution for 16 days, followed by a 16-day elimination period. Our results showed that the liver accumulated the highest Cd concentration (17.41 μg/g), followed by the kidneys (8.00 μg/g) and intestine-stomach (6.68 μg/g), highlighting these tissues as primary targets for Cd accumulation. During the elimination period, Cd concentrations decreased in all tissues, with the head-foot and shell exhibiting over 30% elimination rates. Cd stress also resulted in reduced activities of superoxide dismutase (SOD), catalase (CAT), and glutathione transferase (GST) compared to the control group. Notably, even after 16 days of depuration, the enzyme activities did not return to normal levels, indicating persistent toxicological effects. Cd exposure significantly reduced the diversity of gut microbiota in *P. canaliculata*. Moreover, transcriptome analysis identified differentially expressed genes (DEGs) primarily associated with lysosome function, motor proteins, protein processing in the endoplasmic reticulum, drug metabolism via cytochrome P450 (CYP450), arachidonic acid metabolism, and ECM–receptor interactions. These findings suggest that Cd stress predominantly disrupts cellular transport and metabolic processes. Overall, our study provides comprehensive insights into the toxicological impact of Cd on *P. canaliculata* and emphasizes the importance of understanding the mechanisms underlying Cd toxicity in aquatic organisms.

## 1. Introduction

Cadmium (Cd) is a highly carcinogenic heavy metal, with concentrations of Cd in uncontaminated water typically below 1 ug/L [1] However, both natural phenomena such as volcanic eruptions and anthropogenic activities, including mining, smelting, and fertilization, can dramatically elevate Cd levels by thousands or even tens of thousands of times [2]. As a non-essential toxic element, Cd primarily exists in the aquatic environment in ionic form, characterized by high solubility and mobility, and it accumulates in aquatic organisms through food chain amplification [3,4,5].

Extensive research has been conducted on the effects of waterborne Cd on aquatic organisms. Cadmium can be bioaccumulated in aquatic organisms, leading to adverse physiological and biochemical effects. Research by Yang demonstrated that prolonged exposure to low doses of Cd over four months adversely affects various organs in tilapia, including the gills, muscles, brain, and intestine [6]. This exposure not only increases the prevalence of harmful intestinal microbiota but also impairs the immune function of tilapia and diminishes the liver’s detoxification capacity. Similarly, Wang found that a 30-day exposure to waterborne Cd negatively impacts *Carassius auratus gibelio* by suppressing appetite, which results in reduced growth and survival rates [7]. This exposure also alters the structure and composition of the gut microbiota and disrupts the functional gut barrier. Furthermore, Cd has been shown to compromise the structural integrity of the kidneys, particularly affecting the glomeruli and tubules, as evidenced by Cui [8]. The observed reduction in ATPase activity and abnormal levels of superoxide dismutase (SOD) and catalase (CAT) suggest that Cd interferes with mitochondrial energy metabolism. Despite these known effects, research on the impact of Cd on freshwater invertebrates remains limited. In particular, few studies have thoroughly examined the changes in gut microbiota and transcriptomic responses in these organisms. This lack of comprehensive analysis highlights a significant gap in our understanding of Cd’s impact on invertebrate health. Consequently, there is an urgent need for detailed studies on Cd toxicology in freshwater invertebrates to address this gap.

The gut microbiota is essential for aquatic organism health, as it maintains intestinal barrier integrity, promotes metabolism, and enhances immunity [9]. Waterborne Cd can detrimentally impact the gut of aquatic organisms, leading to alterations in gut length and weight as well as changes in microbial community structure and taxonomic compositions [10]. While the majority of research has concentrated on the toxic effects of Cd on the intestinal tracts of fish, studies on the gut microbiota response of freshwater invertebrates under Cd stress are relatively scarce [6,11,12,13]. Understanding the effects of Cd on these organisms is crucial for assessing overall ecosystem health.

Transcriptomics is the study of gene expression profiles. In recent years, transcriptomics has been widely used to study the effects of heavy metals on aquatic organisms [14]. This approach has helped to deepen our understanding of the potential effects of Cd on the environment and aquatic organisms [15]. For instance, Nair demonstrated that Cd stress induces changes of oxidative enzyme and CYP450, which are related to cellular antioxidant activity and detoxification [16]. Myosin was significantly decreased under Cd exposure, which plays an important role in cell movement and intracellular material transport [17]. Likewise, this technique allowed us to perform transcriptome analysis of apple snails to further reveal how Cd affects apple snails at the molecular level.

In the present study, the apple snail (*Pomacea canaliculata*) was chosen as the indicator species due to its remarkable adaptability and extensive distribution across North America, Africa, and Asia despite its native origin in South America [18]. This species is capable of thriving in a wide temperature range from 10 °C to 35 °C, which contributes to its widespread presence. Notably, the apple snail’s digestive glands have a significant ability to accumulate heavy metals, making it an ideal subject for environmental monitoring. Histopathological examinations have identified substantial changes in the digestive glands under Cd stress, such as increased cupping, degeneration of columnar cells, and narrowing of the lumen [19,20].

This study aimed to investigate the effects of waterborne Cd on apple snails, focusing on enzyme activity, gut microbiota abundance, and transcriptome changes. To achieve this, apple snails were exposed to Cd for 16 days, followed by a depuration period of 16 days. Samples were collected from various tissues, including the heart, intestine-stomach, kidneys, liver, shell, head-foot, and gonads, to provide a comprehensive understanding of Cd accumulation across different parts of the organism. By analyzing changes in Cd accumulation, enzyme activity, gut microbiota, and transcriptome, the study sought to elucidate the mechanisms underlying Cd toxicity in apple snails.

## 2. Results

### 2.1. Cd Accumulation and Elimination in the Tissues of P. canaliculata

**Figure 1:**
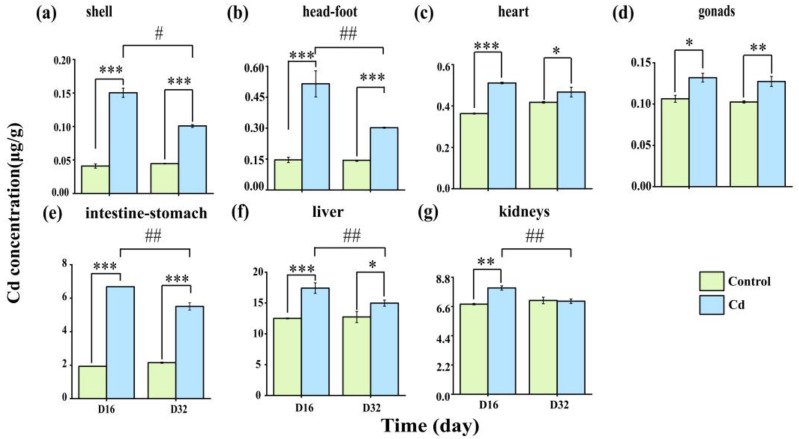
Cadmium accumulation and elimination in the different tissues of *Pomacea canaliculata* on day-16 and day-32. (**a**) Cd concentration in shell; (**b**) Cd concentration in head-foot; (**c**) Cd concentration in heart; (**d**) Cd concentration in gonads; (**e**) Cd concentration in intestine-stomach; (**f**) Cd concentration in liver; (**g**) Cd concentration in kidneys. * indicates a significant difference compared with the control (* *p* < 0.05, ** *p* < 0.01, and *** *p* < 0.01). # *p* < 0.05 and ## *p* < 0.01 are relative to day 32.

Figure 1 shows the accumulation and elimination of Cd in the heart, intestine-stomach, kidneys, liver, shell, head-foot, and gonads of *P. canaliculata*. The results revealed that the accumulation of Cd in various tissues of *P. canaliculata* increased significantly during the exposure period (*p* < 0.05).

Cadmium accumulation was highest in the liver (17.41 μg/g), followed by the kidneys (7.99 μg/g), intestine-stomach (6.68 μg/g), head-foot (0.52 μg/g), heart (0.51 μg/g), shell (0.15 μg/g), and gonads (0.14 μg/g). These levels were approximately 1–3 times higher than those observed in the control group. At the end of the elimination period, significant reductions in Cd concentrations were observed in the intestine-stomach, kidneys, liver, shell, and head-foot in the Cd exposure group compared with those on day 16 (*p* < 0.01). The elimination efficiency of various tissues was as follows: head-foot (40.98%), shell (32.97%), gonads (26.86%), intestine-stomach (17.58%), liver (14.02%), kidneys (12.46%), and heart (8.62%) (Table S1).

### 2.2. Enzyme Activity Response of Snails Under Cd Stress

**Figure 2:**
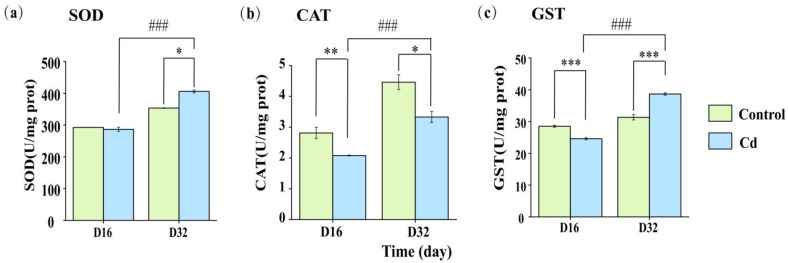
(**a**) Superoxide dismutase (SOD), (**b**) catalase (CAT), and (**c**) glutathione transferase (GST) changes in *Pomacea canaliculata* on day 16 and day 32, respectively. * indicates a significant difference compared with the control (* *p* < 0.05, ** *p* < 0.01, and *** *p* < 0.01). ### *p* < 0.001 is relative to day 32.

On day 16, the activities of superoxide dismutase (SOD), catalase (CAT), and glutathione transferase (GST) in the Cd-exposed group were measured at 286.45, 2.08, and 24.60 U/mg protein, respectively. In contrast, the control group exhibited higher enzyme activities, with values of 292.45, 2.81, and 28.51 U/mg protein, as illustrated in Figure 2. Notably, the activities of CAT and GST in the Cd-exposed group were lower than those in the control group (*p* < 0.05). On day 32, the enzyme activities in the Cd exposure group exhibited significant differences compared with the control group (*p* < 0.05). After 16 days of purification, each enzyme activity had a different reaction pattern. SOD presented no differences on day 16 but had a significant increase in activity on day 32. For CAT, there were significant decreases in activity, compared to the control, for both sampling points, but on day 32, the significance level was different. For GST, significant differences were found for both time periods, but for day 16, the activity of GST was decreased after Cd exposure, while for day 32, there was significant increase in the activity for the snails previously exposed to Cd, as significant differences were still observed between the Cd-treated group and control group.

### 2.3. Characterization of the Gut Microbiota

**Figure 3:**
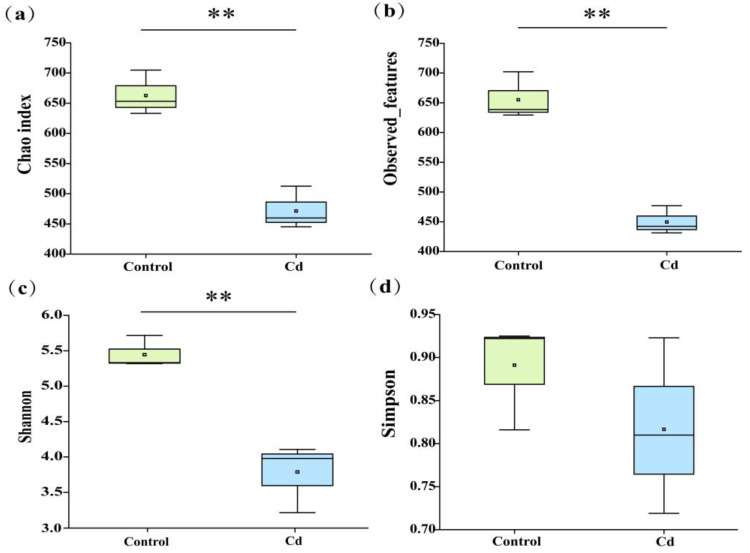
Changes in alpha diversity of *Pomacea canaliculata*. (**a**) Chao index of species richness; (**b**) observed features in the microbiota; (**c**) Shannon index of species diversity; and (**d**) Simpson index of species diversity. ** *p* < 0.01.

The abundance and diversity of the gut microbiota of *P. canaliculata* were assessed by Chao index, observed features, and community diversity indices (Shannon) and Simpson diversity index (Simpson). As shown in Figure 3, Cd stress significantly reduced the Chao index, observed features, and Shannon index (*p* < 0.01), indicating that Cd exposure negatively impacted the diversity of the gut microbiota in *P. canaliculata*.

**Figure 4:**
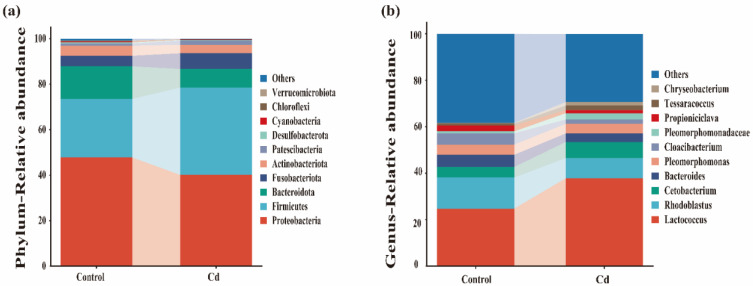
The taxonomic composition of *Pomacea canaliculata* gut microbiota at the phylum (**a**) and genus levels (**b**).

Taxonomic analyses revealed variations in the composition of the gut microbiota of *P. canaliculata*. Proteobacteria, Firmicutes, Bacteroidota, Fusobacteriota, and Actinobacteriota made up the dominant bacterial phyla and accounted for 40.14%, 38.40%, 8.16%, 6.91%, and 3.77% of the relative abundance in Cd-exposed group, respectively. They accounted for 47.77%, 25.75%, 14.35%, 4.6%, and 4.44%, respectively, of the relative abundance in the control group (Figure 4a).

At genus level, the dominant microbiota of the *P. canaliculata* gut microbiota in the Cd-exposed group were *Lactococcus*, *Rhodoblastus*, *Cetobacterium*, *Bacteroides*, and *Pleomorphomonas*, accounting for 37.84%, 8.70%, 6.91%, 3.66%, and 4.15% of the relative abundances, respectively. These proportions were 24.78%, 13.34%, 4.60%, 5.21%, and 4.39%, respectively, in the control group. Compared to the control group, the diversity of *Lactococcus* and *Cetobacterium* increased in the Cd-exposed group, while the diversity of *Rhodoblastus*, *Bacteroides*, and *Pleomorphomonas* decreased (Figure 4b).

### 2.4. Transcriptomic Analysis

We also performed transcriptomic analysis of *P. canaliculata* among the control group and Cd-exposed group through comparative analysis of differentially expressed genes (DEGs) between treated groups. There were a total of 1086 DEGs, in which 478 and 608 unigenes were up-regulated and down-regulated.

**Figure 5:**
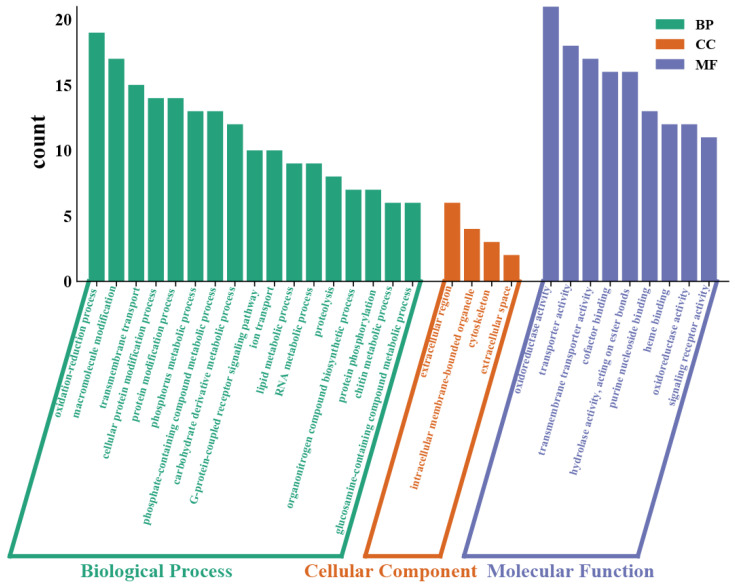
GO annotations of single genes of the transcriptome, where each annotated sequence was assigned at least one of the following GO terms: biological process (BP), cellular component (CC), and molecular function (MF).

**Figure 6:**
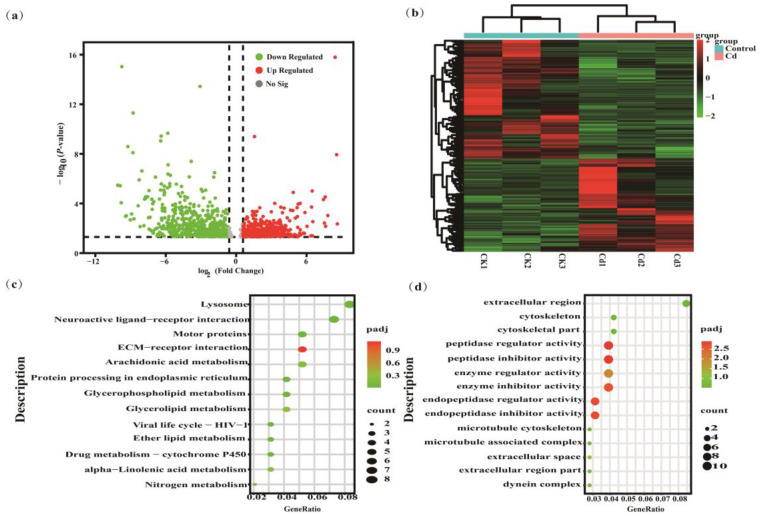
RNA-Seq analysis to identify the differentially expressed genes of *P. canaliculata* upon Cd treatment. (**a**) Volcano plot of DEGs; (**b**) heatmap of DEGs; (**c**) KEGG pathway enrichment analysis of DEGs; (**d**) GO function analysis of DEGs. Pathway enrichment bubble plot colors represent adjusted *p*-values, and bubble sizes represent the number of genes enriched.

To elucidate the functions of these differentially expressed genes (DEGs), they were mapped to the Gene Ontology (GO) database and categorized into three functional areas: biological process (BP), molecular function (MF), and cellular component (CC), comprising 166, 181, and 25 subclasses, respectively, as shown in Figure 5. In the comparison between the 10 μg/L Cd treatment and the control, six GO terms in the BP category were significantly enriched: oxidation-reduction process (GO:0055114), macromolecule modification (GO:0043412), transmembrane transport (GO:0055085), cellular protein modification process (GO:0006464), phosphorus metabolic process (GO:0006793), and carbohydrate derivative metabolic process (GO:1901135), accounting for 14.84%, 13.28%, 11.72%, 10.16%, and 7.81%. Within the MF category, enzyme regulator activity (GO:0030234) and endopeptidase inhibitor activity (GO:0004866) emerged as the most prominent GO terms, representing 3.92% and 3.14%. Additionally, two DEGs were notably enriched in several CC categories, including the extracellular region (GO:0044421) and cytoskeletal part (GO:0044430), accounting for 8.45% and 4.23%, as depicted in Figure 6d.

To further elucidate the pathways affected by Cd exposure in *P. canaliculata*, all DEGs underwent KEGG pathway analysis. As illustrated in Figure 6c, 71 pathways were annotated for the 10 μg/L Cd exposure compared to the control.

## 3. Discussion

### 3.1. Cd Accumulation and Elimination

The accumulation of Cd in mollusks is primarily influenced by biological species, Cd concentration, and exposure duration. In this study, after Cd exposure, all tissues of *P. canaliculata* exhibited significantly higher Cd levels compared to the control group (*p* < 0.05). The highest accumulation was found in the liver, kidneys, and intestine-stomach. Yu observed that in *Mytilus galloprovincialis*, Cd accumulates most in the gills, followed by the digestive glands, and least in the gonads [21]. Similarly, Zhao reported that in *Chlamys farreri*, Cd concentration was highest in the kidneys, followed by the digestive glands, mantle, gills, gonads, and muscle [22]. In *Crassostrea angulata*, Li found that Cd accumulation was highest in the gills, followed by the mantle. In contrast, the adductor muscle showed the least accumulation compared to the other tissues examined [23]. These results indicated that Cd accumulation patterns are species-specific. Additionally, this finding contrasts with previous studies that identified the intestine-stomach as the main organ for Cd accumulation. The discrepancy may be due to exposure duration. Short-term exposure causes Cd to accumulate in the gut mucosal layer, reducing its thickness and mucin content, which prevents transfer to the liver and kidneys [24,25]. Long-term exposure studies have demonstrated that Cd has a high affinity for sulfhydryl groups, enabling it to form Cd-sulfhydryl complexes that enter renal epithelial cells through protein-binding sites. Additionally, Cd activates the protective mechanism of metallothionein (MT) in the liver, forming Cd-MT complexes that are filtered by glomeruli and reabsorbed by proximal tubular epithelial cells. These complexes are then transported to endosomes and lysosomes, where MT is hydrolyzed by proteases, and Cd is taken up by cation transporter proteins [7,26].

Cadmium has a long half-life in mollusks, and its metabolism occurs mainly through excretion. Cadmium removal efficiency is affected by mollusk species, Cd concentration, and tissue recovery ability under Cd stress [27]. In this research, the elimination rates of Cd from various tissues were as follows: cephalopods (40.98%), shells (32.97%), gut (17.58%), liver (14.02%), kidneys (12.46%), heart (8.62%), and gonads (3.48%), indicating tissue-specific variations in Cd elimination. The highest elimination rates in the head-foot and shell were hypothesized to be due to the direct contact with the water [28]. In contrast, Cd elimination in the liver and kidney was only about 10%, which may be due to the redistribution of Cd in the tissues prior to elimination, and as noted by de Conto Cinier this slow excretion rate may be due to the strong binding of Cd to ligands in the liver and kidney [29]. In the present study, we found that the elimination level of Cd in the intestine was higher than that in other organs during the decontamination process, suggesting that unabsorbed Cd is mainly eliminated from the body through the intestine [30].

### 3.2. Effect of Cd Stress on the Enzyme Activity

Cadmium exposure has been demonstrated to impair the oxidase system, resulting in the overproduction of reactive oxygen species (ROS), which subsequently interact with macromolecules such as lipids and proteins. This interaction leads to cellular oxidative stress, apoptosis, and membrane lipid peroxidation [31]. In aquatic ecosystems, cadmium from water sources enters fish and other aquatic organisms through water and food, where it displaces iron ions and induces oxidative stress via the Fenton reaction, causing immune and reproductive disorders [32]. To counteract excessive ROS production, aquatic animals have developed robust antioxidant defenses, primarily involving antioxidant enzymes. Among these enzymes, SOD, CAT, and GST play crucial roles as key antioxidant defense mechanisms. The activities of these enzymes serve as reliable and sensitive indicators for assessing oxidative stress in Cd-exposed aquatic animals. Furthermore, cadmium exposure disrupts mitochondrial membrane potential, promotes the release of cytochrome c, and activates caspase-9 and caspase-3, thereby initiating an intrinsic mitochondria-mediated apoptotic pathway [33,34]. Additionally, a study by Wang found that high concentrations of Cd induced caspase-8 gene expression and activated caspase-3, leading to apoptosis and further damage to the hepatopancreas of bighead carp (*Hypophthalmichthys nobilis*) [35].

Mitochondria are the primary sources of ROS, with complexes I (NADH–coenzyme Q reductase), II (succinate dehydrogenase), and III (coenzyme Q–cytochrome c reductase) in the electron transport chain (ETS) serving as the main sites of ROS production. Under normal conditions, electrons can leak from these complexes, reducing O_2_ to superoxide (O^2−^), which acts as a precursor to hydrogen peroxide (H_2_O_2_), hydroxyl radicals (-OH), and peroxynitrite (ONOO-). Both hydroxyl radicals and peroxynitrite are capable of inducing protein and DNA damage as well as lipid peroxidation [36,37]. SOD serves as the first line of defense against oxidative stress by converting free superoxide to hydrogen peroxide and oxygen [31], while CAT further detoxifies hydrogen peroxide into non-toxic water and oxygen [38]. GST, a family of phase II detoxification enzymes, not only reduces the cytotoxicity of cellular metabolites and environmental chemicals by catalyzing the coupling of reduced glutathione (GSH) to various electrophilic compounds but also participates in the transport of lipids, hemoglobin, and steroids [32,39]. In this study, during the Cd-exposure period, the activities of SOD, CAT, and GST at a concentration of 10 μg/L were lower than those in the control group, aligning with previous research findings [8,40]. This reduction in enzyme activity is likely related to the ability of enzymes in *P. canaliculata* to manage cellular damage caused by a low concentration of Cd (10 μg/L) [41]. After 16 days of purification, the results showed distinct response patterns for each enzyme activity. On day 16, there was no significant difference in SOD activity; however, a significant increase was observed on day 32. This indicates that the snails mitigated oxidative damage caused by Cd stress through the activation of their antioxidant defense system [42]. In contrast, for GST, significant differences were found at both time points. On day 16, GST activity was reduced following Cd exposure, whereas on day 32, GST activity, which is crucial for glutathione (GSH) metabolism, significantly increased. This suggests that GST played a critical role in cellular detoxification during the purification stage, thereby alleviating the toxic effects of Cd stress on the snails [4,43].

### 3.3. Taxonomic Composition Changes of Gut Microbiota

In this study, cadmium stress significantly affect the gut microbiota of *P. canaliculata*. Specifically, alpha diversity indices indicated that 10 μg/L Cd decreased the diversity of gut microbiota in *P. canaliculata*. The results indicated that Cd extensively accumulates in the gut. This accumulation causes the death of bacteria with low tolerance to Cd, significantly reducing microbial diversity within the gut of the apple snails. Ultimately, this change disrupts the stability of the intestinal microbiota, adversely affecting the health of the apple snails. Consequently, it can be inferred that Cd exposure jeopardizes the gut health of *P. canaliculata* by diminishing the diversity of its gut microbiota [12]. The dominant phyla of gut microbiota in *P. canaliculate* are Proteobacteria, Firmicutes, Fusobacteriota, Bacteroidota, and Actinobacteriota, which is consistent with earlier studies [44,45]. The findings further indicated that Cd stress led to a decrease in the abundance of Proteobacteria and an increase in the abundance of Firmicutes and Fusobacteriota. Notably, most pathogenic microbiota belonged to Proteobacteria, while most Firmicutes were beneficial microbiota [46]. Firmicutes are primarily associated with immune regulation, nutrient absorption, and metabolism, whereas Fusobacteriota produce butyric acid under Cd stress, which provides energy and ameliorates gut inflammation [47,48,49].

Moreover, Cd stress stimulated an increase in probiotics such as *Lactococcus* and *Cetobacterium* in the gut microbiota. These probiotics play a crucial role in preventing Cd from entering the body circulation by (1) competitively inhibiting gut absorption of Cd, (2) stimulating gut peristalsis to promote exocytosis of Cd, and (3) improving barrier function [50,51]. Compared with the control, the abundance of *Lactococcus* at the Cd-exposed group was higher, which may be attributed to the negatively charged polysaccharides and membrane proteins on the surface of *Lactococcus* that facilitate Cd adsorption [52]. *Cetobacterium*, commonly found in fish, has also shown beneficial effects. Xie reported that consumption of *Cetobacterium* fermentation products decreased serum lipopolysaccharide (LPS) and diamine oxidase (DAO) activity while promoting the expression of SOD, occludin, and ZO-1, thereby enhancing the gut mucosal barrier in carp [53]. Additionally, previous studies found that *Cetobacterium* produces vitamin B12, a growth factor associated with protein synthesis [10].

In summary, the increased abundance of Firmicutes, Fusobacteriota, *Lactococcus*, and *Cetobacterium* suggests that these bacteria play a crucial role in resisting Cd stress, highlighting adaptive mechanisms involving beneficial microbiota that mitigate the negative effects of Cd stress.

### 3.4. Effects of Cd Exposure on Gene Regulation in the Transcriptome

Cadmium stress promotes cellular oxidative stress and generates large amounts of ROS, necessitating detoxification as a crucial adaptive strategy for *P. canaliculata* to maintain physiological homeostasis. In this study, KEGG pathway enrichment analysis identified several major cellular transport and metabolic pathways, including ECM–receptor interaction, arachidonic acid metabolism, glycerolipid metabolism, alpha-linolenic acid metabolism, and nitrogen metabolism. This suggests that Cd stress triggers the activation of cellular metabolic pathways in *P. canaliculata*. Previous research found that Cd exposure in mice increased alanine transaminase (ALT) and aspartate transaminase (AST) activities, induced high expression of cyclooxygenases (COX-2), caused abnormal lipid metabolism, and elevated arachidonic acid metabolism [54]. Additionally, the findings indicated that most genes are enriched in lysosomes and the CYP450 system. Lysosomes are primarily involved in abnormal cell clearance and energy metabolism. Experiments have shown that Cd affects lysosomes in two ways: it inhibits autophagosome–lysosome binding, leading to the accumulation of autophagosomes and excessive ROS production, which induces oxidative stress in rats; and it promotes the expression of protein hydrolase CTSB, which is associated with lysosome degradation, suggesting that Cd enhances lysosome degradation [55,56].

The CYP450 system, a critical family of enzymes for toxicant metabolism, predominantly exists in the liver and gastrointestinal tract and serves as an essential phase I detoxification enzyme. CYP450 is highly sensitive to heavy metals, and its cysteine residues are highly conserved. Overexpression of CYP450 generates numerous free radical intermediates that can bind to DNA and proteins, inducing cellular damage [42,57,58].

Furthermore, this study found that Cd induces the expression of numerous genes involved in endoplasmic reticulum (ER) protein processing and up-regulates cytoskeleton-related genes. Under Cd stress, the number of misfolded proteins in the ER increases, activating the unfolded protein response (UPR) through three major pathways: ATF6, IRE1α, and PERK-eIF2α. If ER stress (ERS) is not alleviated, the UPR induces apoptosis [59,60].

The cytoskeleton, which consists of microtubules, microfilaments, and intermediate filaments, is also affected by Cd exposure. Templeton demonstrated that short-term exposure to Cd can prevent cytoskeletal disruption and enhance cell survival [61]. This protective effect is mediated through the activation of extracellular signal-regulated kinase (Erk) and the inhibition of Ca^2+^/calmodulin-dependent protein kinase II (CaMK-II). Additionally, the activation of the epidermal growth factor (EGF) receptor and the initiation of the PI3 kinase/Akt pathway contribute to these protective mechanisms. In contrast, prolonged exposure to Cd leads to caspase-independent apoptosis, highlighting the dual nature of Cd’s impact on cellular structures and survival pathways.

## 4. Materials and Methods

### 4.1. Animals and Experimental Conditions

Adult *P. canaliculata* with similar size (7.11 ± 0.072 g) were obtained from a paddy field at South China Agricultural University, Guangzhou (23°9′ N, 113°1′ E). After collection, *P. canaliculata* were washed with tap water to remove sediment and other impurities and incubated for 2 weeks in containers with purified water, and *P. canaliculata* were cultured under following aquaculture environmental conditions: 12 h:12 h light/dark regime, temperature 25 °C, pH of 7.85. During the acclimation period, tap water was changed every two days to maintain optimal water quality, and lettuce constituting 3% of the body weight of the apple snails was provided daily. Ethics approval for snail use and care protocols was granted by the committee of the South China Agricultural University Animal Care and Use (20110107-1).

### 4.2. Experimental Design

Snails of similar size and weight were randomly selected from the same culture environment and divided into two treatment groups: control and Cd-exposed. The Cd-exposed snails were kept in the water with the Cd concentration of 10 μg/L during the initial 16-day exposure period, followed by a 16-day depuration period, during which they were kept in tap water continuously. The 16-day interval was based on the previous research to ensure that snails had a long enough time to accumulate waterborne Cd during the Cd-exposure period [62]. Waterborne Cd usually depends on nearby anthropogenic activities, with low Cd concentrations in affected coastal waters typically ranging from 0.1 to 10 ug/L [63,64,65]. In contrast, the snails in the control group were kept in the tap water. Each group included three replicates, each containing 30 snails. The tested water was changed every two days during the experimental period, and lettuce was supplied daily. The snails were randomly sampled after treatment at day 16 and day 32 post treatment to assess the effects of the treatment. Five snails were taken from each replicate of the treatment and control groups, resulting in a total of 15 snails. These snails were sampled for the following groups: heart, intestine-stomach, kidneys, liver, shell, head-foot, and gonads, the samples of which were frozen immediately in liquid nitrogen and stored in a −80 °C refrigerator for the next analysis.

### 4.3. Enzyme Activity Assay

About 0.5 g of five snails’ liver from each group were ground in liquid nitrogen using a mortar, and 4.5 mL of cold saline was added during grinding for rapid homogenization; each treatment consisted of three sets of replicates. The liver was selected as the target tissue because it is the main organ for accumulation and metabolism [66,67]. The homogenate was centrifuged at 3000 rpm for 10 min at 4 °C. The supernatant obtained after centrifugation was used immediately for the determination of biochemical parameters. These parameters included SOD, CAT, and GST.

These biochemical parameters were analyzed using assay kits purchased from Nanjing Jiancheng Bioengineering Institute, Nanjing, Jiangsu Province, China. Detailed procedures for the measurement of biochemical parameters can be found in the Supplementary Material Text S1.

### 4.4. Cd Concentration Determination

About 0.05–0.1 g of the powder was weighed, and 10 mL of HNO_3_ was added and digested using a microwave digestor (Mars 6; CEM Corporation, Matthews, NC, USA). A blank method was used during the digestion of each batch to eliminate the interference of reagents and solvents. For quality control, standard shrimp material (GBW10050a; Institute of Geophysical and Geochemical Exploration, Chinese Academy of Geological Sciences) was digested under the same conditions to ensure that the recoveries of tissues were controlled at 95–105%. Cadmium concentrations were determined for each tissue using ICP-MS, and Cd concentrations were expressed as μg-g^−1^ dry weight (*w*/*w*).

### 4.5. Analysis of Gut Microbiota

To investigate the effects of Cd exposure on the composition of the snail microbiota, we performed 16S rDNA gene sequencing analysis. Genomic DNA was extracted from the samples using sodium dodecyl sulfate (SDS), and its purity and concentration were assessed by gel electrophoresis. After quantification with Qubit, the amplified V3–V4 regions were sequenced on the Illumina HiSeq platform. The library was constructed using the NEB Next Ultra DNA Library Preparation Kit. Microbial diversity was analyzed using the Chao index, observed features, Shannon, and Simpson indices calculated with QIIME 2 software (version 2024.10).

### 4.6. RNA Extraction and Transcriptome Analysis

Total RNA was isolated from different groups of tissues using TRIzol kits (Invitrogen, Carlsbad, CA, USA). The RNA was initially quantified using a Qubit 2.0 Fluorometer (Invitrogen, Thermo Fisher Scientific, Waltham, MA, USA), and its integrity and total amount were assessed with an Agilent 2100 bioanalyzer (Agilent Technologies, Palo Alto, CA, USA). The raw data obtained were then filtered using fastp software (Version 0.24.0). Subsequently, the bipartite sequences were aligned with the reference genome using Hisat2 (v2.0.5). Differential expression analysis of the genes was conducted using DESeq2 (1.20.0) software, with the criteria set as |log2(FoldChange)| ≥ 1 and padj < 0.05. Furthermore, Gene Ontology (GO) and Kyoto Encyclopedia of Genes and Genomes (KEGG) enrichment pathway analyses were performed using cluster Profiler software (3.8.1).

### 4.7. Statistical Analysis

To ensure the accuracy and reliability of the experiment, three replications were conducted, and the data were analyzed using IBM SPSS Statistics27.0. A normality test was performed through Shapiro–Wilk test. The data used in the experiment were confirmed to meet the assumptions of normality. Differences between treatments were assessed using Student’s *t*-test. Equality of variances were determined using the Levene test. Data were averaged from two independent experiments and are shown as means ± standard deviations (S.D.). * represents differences compared to the control group (*p* < 0.05).

The elimination rate (%) is the percentage decrease in the initial value (the 16 days of exposure), and the following definition was used:

Elimination rate = (C_elimination_ − C_exposure_)/C_exposure_ × 100%

C_elimination_ is the concentration of Cd in each tissue after a 16-day elimination period; C_exposure_ is the concentration before elimination [28].

## 5. Conclusions

This study investigated the effects of Cd exposure on *P. canaliculata*, focusing on Cd accumulation, enzyme activity, gut microbiota, and transcriptome analysis. Our findings confirmed that Cd accumulates in various tissues, including the liver, kidneys, intestine-stomach, head-foot, heart, shell, and gonads, with the highest concentrations observed in the liver, followed by the kidneys and intestine-stomach. In terms of elimination efficiency, the head-foot exhibited the greatest capacity, followed by the shell and intestine-stomach. Under Cd stress, *P. canaliculata* showed reduced activities of SOD, CAT, and GST compared to the control group, and these enzyme activities did not return to normal levels during the depuration period. Furthermore, Cd stress led to a reduction in the diversity of gut microbiota. Specifically, exposure to low concentrations of Cd resulted in an increased abundance of Firmicutes and Fusobacteriota while reducing the abundance of Proteobacteria, Bacteroidota, and Actinobacteriota. Transcriptome analysis further revealed that Cd exposure affects cellular transport and metabolism. These findings enhance our understanding of the chronic toxicological effects of Cd on *P. canaliculata* and provide valuable insights into the accumulation and metabolic processes of Cd in aquatic invertebrates.

## Data Availability

Guikui Chen should be contacted to request data from this study.

## Supplementary Materials

The following supporting information can be downloaded at: https://www.mdpi.com/article/10.3390/ijms26020751/s1.
