# Supplementary material for: Toxicology Effects of Cadmium in Pomacea canaliculate: Accumulation, Oxidative Stress, Microbial Community, and Transcriptome Analysis
Source: Int J Mol Sci. 2025 Jan 17;26(2):751. doi: 10.3390/ijms26020751 (PMC11766043; doi:10.3390/ijms26020751)
Supplement: Supplementary file 1 [file ijms-26-00751-s001.zip › ijms-3384921-supplementary.pdf]

International Journal of
Molecular Sciences

MDPI

Supplementary

Toxicology effects of cadmium in *Pomacea canaliculate*: accumulation, oxidative stress, microbial community, and transcriptome analysis

Mingxin Qiu [a], Xiaoyang Bi [a], Yuanyang Liu [a], Huashou Li [a], Dongqin Li [b]* and Guikui Chen [a]*

a Guangdong Laboratory for Lingnan Modern Agriculture, Guangdong Provincial Key Laboratory of Agricultural & Rural Pollution Abatement and Environmental Safety, College of Natural Resources and Environment, South China Agricultural University, Guangzhou 510642, China
b Institute of Quality Standard and Monitoring Technology for Agro-products of Guangdong Academy of Agricultural Sciences, Guangzhou 501640, China
* Correspondence: author: lidongqin@gdaas.cn; guikuichen@scau.edu.cn

Contents

Table S1 Cadmium accumulation and elimination rates among the main tissues after treatment with cadmium in *Pomacea canaliculate.*

Text S1. Measurement of oxidative activity of cadmium in *Pomacea canaliculate*.

Table S1. Cadmium accumulation and elimination rates among the main tissues after treatment with Cadmium in *Pomacea canaliculate.*

Tissues	Cadmium concentration (μg/L)		Elimination rates (%)
	Time (16d)	Time (32d)	
heart	0.511	0.467	8.62
intestine-stomach	6.683	5.508	17.58
kidneys	7.993	6.996	12.46
liver	17.413	14.973	14.02
shell	0.150	0.101	32.97
head-foot	0.515	0.304	40.98
gonads	0.138	0.101	26.86

Text S1. Measurement of oxidative activity of Cadmium in snails

SOD activity analysis

According to the instructions provide by SOD assay kit, 20 μL of the supernatant was combined with 20 μL working solution of xanthine oxidase, as well as 200 μL of the water-soluble tetrazolium salt solution (WST-1). The resulting mixture was thoroughly mixed and incubated for 20 min at 37 °C. Following the incubation period, the enzyme activity was measured at 450 nm.

CAT content analysis

According to the CAT assay kit instructions, 0.1 mL of supernatant, 1 mL of Reagent I, and 0.1 mL of Reagent Ⅱ were combined and thoroughly mixed. The mixture was incubated for 60 seconds at 37 °C. Subsequently, 1 mL of Reagent Ⅲ and 0.1 mL of Reagent Ⅳ were added immediately. The enzyme activity was then measured at 405 nm.

GST level analysis

According to the instructions provided by the GST assay kit, a well containing 0.3 mL of base, 1 mL of regent B, 1 mL of absolute alcohol and 1 mL of supernatant was mix uniformly. Then, the sample was centrifuged at 4000 rpm for 10 min. 2 mL supernatant was added with 2 mL of regent C and 0.5 mL of regent D. The mixture was thoroughly mixed and incubated for 15 min. Subsequently, the GST level was measured at 412 nm.
